# “The Italian Study on Recovery 2” Phase 1: Psychometric Properties of the Recovery Assessment Scale (RAS), Italian Validation of the Recovery Assessment Scale

**DOI:** 10.3389/fpsyt.2019.01000

**Published:** 2020-02-05

**Authors:** Ileana Boggian, Dario Lamonaca, Marta Ghisi, Gioia Bottesi, Alessandro Svettini, Luigi Basso, Katia Bernardelli, Silvia Merlin, Robert Paul Liberman, S.I.R. 2 group, Ileana Boggian

**Affiliations:** ^1^ Center for Mental Health, Psychiatry 3, AULSS 9 Scaligera, Verona, Italy; ^2^ Department of Psychology, University of Padua, Padua, Italy; ^3^ Department of Mental Health, ASP Agrigento, Agrigento, Italy; ^4^ Psychiatric Service, Health Trust of Bolzano, Bolzano, Italy; ^5^ UCLA Psych REHAB program, University of California, Los Angeles, Los Angeles, CA, United States

**Keywords:** Recovery Assessment Scale, validation, recovery, Study on Recovery, Psychometric Properties of the R.A.S

## Abstract

**Background:**

The achievement of recovery is related to the notion of developing personal potential and restoring a legitimate social role, even against the backdrop of mental illness limitations. It is still difficult to fully understand this highly subjective and dynamic process. Therefore, in order to test the recovery process, specific tools, still only marginally used in our country, are needed.

**Aims:**

The Italian Study on Recovery is the first study aimed at confirming the validity of the Italian version of the Recovery Assessment Scale (RAS), an instrument developed with the goal of detecting recovery among patients.

**Method:**

This multicentric research involved several Mental Health Services from various parts of Italy. The first phase of the study consisted in the administration of the Italian translation of RAS, previously used in a pilot study conducted in 2009. RAS was administered to 219 patients diagnosed with psychosis, whose mental disorder lasted for at least 5 years.

**Results:**

Findings supported the good psychometric properties of the Italian version of RAS, demonstrating its capability of identifying patients matching the “in recovery” operational criteria.

**Conclusions:**

In consideration of the results highlighting the good psychometric properties of RAS, the present study may contribute to the diffusion of instruments to be included in Mental Health Service planning in the Italian context, in order to start a recovery-oriented transformation.

## Introduction

In scientific literature there is a growing consensus in defining mental health as an ongoing interactive process, a personal journey to recover the sense of self, the ability to self-manage the illness, and a sense of belonging and restoring one’s own community life (1). The concept of *recovery* originates from the consumer movement of the ‘70s and ‘80s, and continues to be used and developed internationally by people with experiences of mental illness (2). Data from prospective and qualitative studies, as well as from service users accounts (3–6) also contributed creating a new sensibility on this subject, focusing also on the healing factors involved in the process of recovery from mental illness and influencing the organization of models of mental health services (7). The concept of recovery has also been described as the guideline to transform the mental health system (8). Scientific literature emphasized the key role that organizational culture at all levels of mental health services has in facilitating a new recovery-oriented approach (9). For recovery to be fully integrated into clinical practice, an environment that embraces recovery ideals is essential. The principles of recovery must be integrated in all organizational processes (10).

As such, a practice that becomes *recovery-oriented* identifies a mental-health approach that incorporates self-determination and individualized care as founding principles. Values such as hope, social inclusion, goal setting, and patient self-management become particularly relevant through this approach. These principles should permeate the standards of treatment (11). Therefore, the notion of recovery means moving away from a superficial understanding of mental illness as a pathology, or simplistically assuming care is a process aimed at maintenance or stabilization of the patient’s mental health. Recovery is a holistic approach to wellness aiming at and founded on individual strengths, partnership, and change (12). However, if, on the one hand, it is increasingly important that professionals have a clear understanding of the principles of recovery, on the other hand, there is no universal agreement about what recovery really means in the daily context, and by the lack, in Italy at least, of validated instruments to measure recovery (13).

The Australian Mental Health Outcomes and Classification Network (14) identified as many as 22 instruments designated to measure recovery and 11scales developed to assess the degree of recovery orientation in the provision of services. When choosing an instrument for such purposes, it has to be short and easy to use, with good psychometric properties, able to get the users’ perspective and to assess domains related to personal recovery. With these characteristics, the available instruments measuring personal recovery are: the *Recovery Assessment Scale* [RAS, (15)], the *Illness Management and Recovery Scale* [IMR, (16)], and the *Stages of Recovery Instrument* [STORI, (17)]. Some instruments are available to assess the degree of inclusion of recovery-oriented practices in the health-care system: the *Recovery Oriented System Indicators Measure* [ROSI, (18)], the *Recovery Self-Assessment* [RSA, (19)], the *Recovery Oriented Practices Index* [ROPI, (20)], and the *Recovery Promotion Fidelity Scale* [RPFS, (21)]. A complete collection of 33 of such instruments is available in the compendium of recovery and recovery-related instruments (22).

The RAS is a tool designed for the assessment of recovery in psychiatry (23). The first version of this scale was developed by Giffort et al. (24); it consisted of 39 items derived from the analysis of four stories of recovery of people with severe mental illness. The same items were then reviewed by a group of 12 patients, using the technique of focus groups, which expanded the items to 41 and effectively defined the scale’s final version. A factor analysis conducted on the administration of this final version to a sample of 1,824 patients identified five factors, which suggested the grouping of the items in the following subscales: *personal confidence and hope* (Factor 1); *willingness to ask for help* (Factor 2); *goal and success orientation* (Factor 3); *reliance on others* (Factor 4); and *not being dominated by symptoms* (Factor 5) (15). These data were further analyzed, suggesting that the RAS total score could be related to several measures of social functioning and to the severity of symptoms. In fact, the data demonstrated the existence of a direct relationship between the RAS total score and quality of life, showing good convergent validity, as well an inverse relationship between self-reported psychiatric symptoms and recovery, presenting a good divergent validity. These five factors can be related to the four domains of recovery identified by Ralph (25): Factor 1 corresponds to the internal factors related to confidence and self-determination; Factors 2 and 4 can be connected to external factors such as the readiness to trust others and ask for help; Factor 5 is equivalent to the self-managed care, i.e., the ability to manage the illness; Factor 3, linked to life goals and being an enterprising person, seems to correspond to empowerment. These results suggest that RAS may measure five critical domains, corresponding to the main recovery-related processes. It is worth noting that hope, the construct measured by the Herth Hope Index (26, 27), was highly correlated with all five factors, demonstrating that it represents an essential element in the process of recovery (15). It is thereby possible to assert that the factors associated with recovery evaluated by RAS represent a complex set of constructs whereby each factor is associated with more than one construct and each construct to more than one factor. A study conducted by Mukolo et al. (28) also showed a strong association between this complex concept of recovery and self-esteem, suggesting that the RAS score might be a strong predictor of the Rosenberg Self-Esteem scale score [RSE, (29)]. In this research, indeed, RAS was found to be associated with the overall positive self-esteem. However, not all the RAS domains were related to self-esteem: while the scores of subscale 1 “personal confidence and hope” were significantly associated with positive self-esteem, Factor 2 “willingness to ask for help” (which measures the person’s ability to get help from others in times of trouble) and Factor 4 “reliance on others” (assessing the disposition to count on others) were on the contrary associated with external factors. Factor 3 “goal and success orientation” and Factor 5 “not being dominated by symptoms” did not seem related to the construct of self-esteem, at least based on what emerged from this study of Mukolo et al.

A preliminary research designed to test the psychometric properties of the Italian version of RAS (30) showed good internal consistency for the total score (Cronbach’s α = 0.92) as well as for the single subscales, with values ranging between acceptable (α > 0.53) and excellent (α > 0.83). Based on the operational criteria of recovery (31), patients involved in this study were divided into two groups (“in recovery” or “not in recovery”). Through the receiver operating characteristic (ROC) curve analysis, it was also possible to find a cutoff score of 160 (range 41–205) for subjects in recovery, not yet found in the previous studies using the English version of RAS. In addition, univariate analysis of variance (ANOVA) demonstrated that the two groups of patients differed in all the RAS subscales, except for subscale 4 “reliance on others.” In this preliminary study, therefore, the Italian version of the RAS scale showed a good ability to discriminate between patients in recovery and those who are not, confirming the results of the study of Corrigan et al. (15) and of the Australian group of McNaught et al. (32). However, this preliminary research presented some limitations: the small number of the sample, its non-representativeness, and the lack of co-administration of other rating scales to correlate with the scores obtained with RAS.

Unlike other countries, like Australia or Japan, where some research had been conducted focusing on instruments evaluating recovery paths in mental health (32, 33), in Italy there were very few studies on this subject (34). The aim of this research was to confirm and extend the preliminary results of the research previously conducted with the Italian version of RAS (30), overcoming the abovementioned limitations. The present study, therefore, aims at further investigating and validating the psychometric properties of the Italian version of RAS, in order to have a valid and specific tool to be used in the organization of recovery-oriented services.

In addition to the evaluation of internal consistency of subscales and of intercorrelations between them, the study evaluated the convergent validity between the subdomains measured by RAS and other constructs such as self-esteem, empowerment, quality of life, and social functioning. In particular, based on the already mentioned work of Mukolo et al., the study investigated the associations between the following subscales:

1) “Personal confidence and hope” and a measure of self-esteem
3) “Goal and success orientation” and empowerment measures
5) “Not being dominated by symptoms” and an index designed to evaluate the impact of symptoms on the life of the patient

No specific hypotheses were formulated for the remaining subscales: 2, “willingness to ask for help,” and 4, “reliance on others.”

Furthermore, in line with what was reported by Corrigan et al. (15), a positive association was explored between the total score of RAS and a self-evaluated measure of quality of life, as well as a negative correlation between the RAS total score and symptoms as self-reported by patients.

Finally, another task of the study was to examine the discriminative power of RAS, testing more specifically the utility of a cutoff score identified by the ROC curve analysis, in order to understand the degree to which extent this instrument could correctly identify users in recovery, in compliance with standards already established in the literature. (31).

## Materials and Methods

### Participants

A total of 219 patients (of which 37.2% were women) entered the study: they were recruited in 25 mental health services (day centers, outpatient mental health centers, in- and outpatient psychiatric rehabilitation centers) from various parts of Italy. Each service had to recruit a minimum of 10 patients, at least 40% of them matching the UCLA criteria for recovery (31). More specifically, these criteria consisted of: a period of 2 years or more with symptom remission, full- or part-time involvement in work or school, independent living without supervision by family or surrogate caregivers, not being fully dependent on financial support from disability insurance, and having friends with whom activities are shared on a regular basis. The study was conducted following the principles of research ethics, in accordance with the Declaration of Helsinki. All participating investigators were asked to act within this ethical frame. Due to the observational nature of the study, an ethics review procedure was not performed. All participants provided a written informed consent for potential research analysis and anonymous reporting of ﬁndings in aggregate form, in accordance with the Italian legal and ethical requirements. All participants were informed in detail about the aims of the study, the voluntary nature of their participation, and their right to withdraw from the study at any time and without being penalized in any way. No specific protocol was followed to protect patients from potential harm: investigators, though, were with patients during the whole interview procedure.

Participants ranged from the age of 24 to 65 (M = 44.62; SD = 9.04), and the length of their involvement in the educational system ranged from 3 to 25 years (M = 11.28; SD = 3.35). The age of onset of the disorder varied between 10 and 50 years (M = 25.68; SD = 8.76), and the duration of the psychopathology was between 1 and 40 years (M = 18.08; SD = 8.93). Additionally, 82.2% (N = 180) of participants reported not to have had previous psychiatric hospitalizations; 74% individuals declared not to suffer from other pathologies at the time of the assessment.

Participants were divided into two subgroups, based on matching or not the UCLA operational criteria of recovery, as evaluated by investigators. Sixty-five patients formed the “recovery” group, whereas 151 comprised the “not in recovery” one. Due to lack of information, it was not possible to assess the recovery status of 3 patients; therefore, the final sample involved in the present research consisted of 216 participants.

### Self-Report Measures

*Recovery Assessment Scale* [RAS; (15); Italian translation by (30)]. This is a self-report measure consisting of 41 items rated on a five-point Likert scale (1 = “strongly disagree”; 5 = “strongly agree”). It assesses five dimensions of the recovery process: “personal confidence and hope” (nine items), “willingness to ask for help” (three items), “goal and success orientation” (five items), “reliance on others” (four items), and “no domination by symptoms” (three items). The total score results from the answers to all items. In its original version, RAS showed good psychometric properties. Internal consistency for the five RAS factors varied between α = .52 (Factor 2, “willingness to ask for help”) and α = .83 (Factor 1, “personal confidence and hope”). As previously described, the Italian adaptation of the RAS had preliminarily shown good psychometric properties as well (30).

*Empowerment Scale* [SESM; (35); Italian version by (36)], also known as SESM, consists of 28 items to be rated on a four-point Likert scale (1 = “strongly agree,” 4 = “strongly disagree”), investigating what people think about life and how they make decisions. This scale measures constructs such as empowerment, self-esteem, self-efficacy, and personal value. Evaluated on a sample of 35 “severe” psychotic patients, it showed good internal consistency (α = .86) as well as good temporal stability [intraclass correlation coefficient (ICC) = .75)]. Discriminant validity also resulted as satisfying (37). The Italian version is characterized by good psychometric properties: internal consistency resulted as α = .81, and temporal stability was ICC = .93 (36).

*Rosenberg Self-Esteem* [RSE; (29); Italian version by (38)] is a questionnaire that consists of 10 items rated on a four-point Likert scale (1 = “strongly disagree,” 4 = “strongly agree”) assessing the overall self-esteem and taking into account also the individuals’ weaknesses. It may be used to evaluate the effectiveness of rehabilitation programs (35, 39–41). Several studies examined the psychometric properties of RSE and reported α coefficients varying between.72 and.88 (42). The Italian version shows good internal consistency (α = .84), acceptable 2-week temporal stability (r = .76), and good construct validity (38).

*Manchester Assessment Quality of Life* [MANSA; (43, 44); Italian translation by (45)]. This is the brief and modified version of the *Lancashire Quality of Life Profile* [LQLP; (46)], designed to evaluate the quality of life of psychiatric patients. The questionnaire consists of 17 items; 13 of them assess the individuals’ satisfaction as regards different aspects of life, using a seven-point Likert scale (1 = “It couldn’t be worse than like this,” 7 = “It couldn’t be better than like this”): four items require a three-level evaluation (“yes,” “no,” “I don’t know”). The original version showed good psychometric properties: internal consistency resulted as α = .74, and the correlation between MANSA and LQLP was r = .83, thus indicating good convergent validity. Furthermore, divergent validity was good: the Pearson’s correlation with the Brief Psychiatric Rating Scale [BPRS; (47)] was r = −.49, a result consistent with those previously reported in literature (48–50). The Italian version employed in the present research is the translation performed by Ruggeri (1998); to date, no studies validating the Italian MANSA have been conducted.

*Health of the Nation Outcome Scale—Roma* [HoNos; (51); Italian version by (52, 53)]. This instrument assesses both psychopathological and social functioning aspects of psychiatric patients; it has to be completed by professionals who collect information from patients, their relatives, and other professional care-takers. Each of the 18 items can be rated between 0 (“absence of problem”) and 4 (“severe problem”), and it is possible to perform six evaluations of the same patient across time. The total score varies between 0 and 72: the higher the score, the worse the individual’s functioning. Internal consistency of the Italian version is satisfying (α = .70), better than what reported by Orrell et al. (54) and Wing et al. (51, 55).

### Statistical Analyses

Chi-squared and univariate ANOVAs were conducted to compare the two groups (“recovery” and “not in recovery”) in relation to socio-demographic variables and the RAS subscales scores.

Cronbach’s alpha (α) was computed to assess the internal consistency of RAS subscales, whereas Pearson’s correlations were calculated for analyzing both intercorrelations among the RAS subscales and convergent/divergent validities.

Lastly, the analysis of the ROC curve was performed to identify a cutoff score; positive agreement (patients evaluated as “in recovery” by investigators and who scored higher than the RAS cutoff) and negative agreement (patients evaluated as “not in recovery” by investigators and who scored lower than the cutoff on RAS) percentages were also computed.

## Results

The two groups did not differ with regard to the following socio-demographic variables (**Table 1**): gender (χ^2^_(1)_ = 0.14; p = .71); marital status (χ^2^_(4)_ = 1.00; p = .91); age (F_(1,214)_ = .44; p = .51); education (F_(1,214)_ = 2.01; p = .16); age of onset of the disorder (F_(1,214)_ = .92; p = .34); duration of the disorder (F_(1,214)_ = 2.90; p.09); and co-occurrence of other disorders (χ^2^_(1)_ = .01; p = .98).

**Table 1:** Sociodemographic variables between the two groups (percentages).

	In recovery (N = 65)	Not in recovery (N = 151)	Total (N = 216)
Living condition			
Alone	29.7%	15.1%	19.5%
Family	40.6%	41.8%	41.4%
In-law family	10.9%	6.8%	8.1%
Supported living condition	17.2%	34.9%	29.5%
Other	1.6%	1.4%	1.4%
Occupation			
Unemployed	3.4%	60.0%	43.4%
Employed	53.4%	15.7%	26.8%
Supported employment	37.9%	23.6%	27.8%
Student	0%	.7%	0.5%
Other	5.2%	.0%	1.5%
Social Relationships			
Social isolation	0%	18.8%	13.3%
Acquaintances	0%	43.6%	31.0%
A few real friends	47.5%	26.2%	32.4%
Friends on a regular basis	52.5%	11.4%	23.3%
Autonomy			
Dependent from others	0%	2.0%	1.4%
A few difficulties in different functional areas	0%	43.0%	30.1%
Normal	100.0%	55.0%	68.5%

Unsurprisingly, the two groups showed significant differences as regards: living condition (χ^2^_(4)_ = 10.56; p = .03); occupation (χ^2^_(4)_ = 62.71; p < .001); social relationships (χ^2^_(3)_ = 75.43; p < .001); and autonomy (χ^2^_(2)_ = 47.72; p < .001) (**Table 1**).

### Internal Consistency

Internal consistency of the RAS subscales ranged between acceptable and excellent values (**Table 2**). The RAS total score also showed excellent internal consistency (α = .93) (**Table 2**).

**Table 2:** Internal consistency (Cranach’s α) and intercorrelations between the RAS scales.

RAS Subscales	α	2	3	4	5	Total Score
1. Personal confidence and hope	.80	.38^**^	.70^**^	.38^**^	.55^**^	.90^**^
2. Willingness to ask for help	.70		.39^**^	.31^**^	.35^**^	.58^**^
3. Goal and success orientation	.81			.38^**^	.45^**^	.82^**^
4. Reliance on others	.60				.30^**^	.60^**^
5. No domination by symptoms	.66					.70**

** = p < .001.

In our sample, internal consistency of the SESM scale proved to be good also in the present sample (α = .73), as well as for RSE (α = .81), MANSA (α = .84), and HoNos (α = .83).

### Intercorrelations Between Subscales

Intercorrelations between the RAS subscales were all positive and statistically significant; *r*s values resulted in the medium–large range, thus indicating that the five subscales measure different aspects of the same construct (**Table 2**).

### Convergent and Divergent Validity

The subscales “personal confidence and hope,” “willingness to ask for help,” “goal and success orientation,” and “no domination by symptoms,” as well as the RAS total score, showed positive and medium–large correlations with the total scores of RSE, SESM, and MANSA. Furthermore, the “goal and success orientation” subscale showed a negative weak correlation with the HoNos total score. Similarly, the “no domination by symptoms” subscale resulted as negatively and weakly correlated with the HoNos total score. It is worth noting that the “reliance on others” subscale showed overall weak associations with all the investigated constructs (**Table 3**).

**Table 3:** Convergent and divergent validity of RAS.

RAS Subscales	RSE	SESM	MANSA	HoNos
1. Personal confidence and hope	.64**	.66**	.59**	-.13
2. Willingness to ask for help	.39**	.41**	.17*	-.13
3. Goal and success orientation	.52**	.58**	.45**	-.20**
4. Reliance on others	.23**	.29**	.20**	.03
5. No domination by symptoms	.46**	.43**	.44**	-.17*
RAS total score	.65**	.67**	.17*	.57**

RSE, Rosenberg Self-Esteem; SESM, Empowerment Scale; MANSA, Manchester Assessment Quality of Life; HoNos, Health of the Nation Outcome Scale–Roma. *p < .05; **= p < .001.

### Differences Between Groups

As shown in **Table 4**, participants included in the “recovery” group obtained significantly higher scores than those in the “not in recovery” group in all the RAS subscales; the only exception was represented by the “reliance on others” subscale, where no difference between groups resulted.

**Table 4:** Means (SD) obtained by the two groups on the RAS subscales.

RAS Scale	In recovery (N = 65)	Not in recovery (N = 151)	F_(1.216)_	p
1. Personal confidence and hope	35.44 ( ± 5.09)	32.56 ( ± 4.91)	15.19	< .001
2. Willingness to ask for help	12.72 ( ± 1.81)	12.07 ( ± 1.87)	5.21	.02
3. Goal and success orientation	21.55 ( ± 2.46)	19.68 ( ± 3.05)	19.03	< .001
4. Reliance on others	16.62 ( ± 2.35)	15.99 ( ± 2.26)	3.30	.07
5. No domination by symptoms	11.64 ( ± 2.12)	10.48 ( ± 2.56)	10.09	.002
RAS total score	167.24 ( ± 18.17)	155.19 ( ± 18.38)	19.50	< .001

### ROC Curve Analysis

The analysis of the ROC curve allowed to identify 158 as the optimal score in discriminating patients “in recovery” from those “not in recovery.” Four participants were not included in the analyses since they omitted more than one-third of the RAS items, making it impossible to compute the RAS total score. The not-parametrically-computed area below the curve resulted wide (.70). By assuming 158 as the cutoff score, the RAS sensibility is 78.5% and specificity 61.2%. Therefore, 51 out of 65 patients were correctly classified as “in recovery” and 90 out of 147 were correctly classified as “not in recovery.”

## Discussion and Conclusions

By repeating and expanding a preliminary study on the same topic, the present one was conducted to further investigate and confirm the psychometric properties of the Italian translation of RAS in order to validate it. The results support and extend what had already emerged in the research of Boggian et al. (30), confirming the good psychometric properties of the preliminary Italian version of the RAS. On the basis of these results, this approach can therefore be used in routine clinical practice as a tool for tracking progress of the service user, and as a tool for scheduling and assessing the effectiveness of mental health services.

In line with the previous preliminary findings, the internal consistency was found good both for the single subscales and for the entire scale. In addition, Cronbach’s α of subscale 4 “reliance on others,” even if it not reaching optimal values, proved to be acceptable in the present study (α = 0.60, compared to α = 0.53 in the 2011 study). Intercorrelation values between subscales observed in this sample were also consistent with those reported in the previous research, further confirming that the diverse dimensions investigated by RAS measure constructs that are similar but not entirely overlapping.

RAS was found to be a questionnaire with good convergent validity. In particular, the constructs “personal confidence and hope,” “willingness to ask for help,” “goal and success orientation,” and “not being dominated by symptoms” correlated strongly with high levels of self-esteem, empowerment, and quality of life. Differently from what found by Mukolo et al. (28), who had assumed and observed specific relationships between, on the one hand, self-confidence and self-esteem, and, on the other hand, between success orientation and empowerment, the results of this research showed that the different dimensions of recovery are associated with increased positive self-esteem and empowerment. Additionally, the study revealed the strong correlations between the different dimensions investigated by RAS and the measure of quality of life, and therefore of the subjective well-being as reported by the patient with a psychiatric disorder. The same considerations are also valid for the RAS total score. Furthermore, in line with what Corrigan et al. (15) had already observed, the total score is strongly correlated with quality of life. In addition, there was a weak but significant negative relationship between, on the one hand, the presence of self-reported symptoms, as measured by the HoNos scale, and, on the other, the score on the subscale “not being dominated by symptoms” and the total of the RAS.

Confirming what had already emerged in the previous research of Boggian et al. (30), the comparison of the various RAS subscales scores of subjects assessed by clinicians as “in recovery” with those of patients “not in recovery” showed that the former were more self-confident, more willing to ask for help, more goal and success oriented, and with a stronger perception of not being dominated by symptoms than participants who were “not in recovery.” In line with the prior Italian research, these two groups did not differ in the subscale assessing the tendency to trust other people. This result, jointly taken with the low internal consistency of subscale 4 “reliance on others” and its low convergent validity with other constructs similar to recovery, seems to suggest that this subscale represents a weakness of RAS.

As regards the discriminative power of this instrument, results demonstrated a good discriminating power of the cutoff score (158) identified in this study, with a sensibility of 78.5% and a specificity of 61.2%. Through the RAS score, it was possible to correctly identify subjects in recovery from those not in recovery, with a low number of false negatives (i.e., subjects not identified as in recovery through the RAS cutoff score but in recovery as evaluated by the clinician). However, this instrument tends to produce a number of false positives (about one-third of patients with a RAS score >158 but assessed as not in recovery by clinicians). It seems that RAS, as a self-assessed standardized measure of recovery, tends to overestimate the number of subjects effectively in recovery. Similar results were obtained in the preliminary study of Boggian et al. (30), which, based on a cutoff score of 160, presented a sensibility of 77.3% and a specificity of 65.9%. Therefore, given the limits represented by the number of false positives, using the cutoff score of 158, it is possible to enhance the sensitivity of this instrument (and consequently the identification of patients actually in recovery). It is also important to consider that the comparison criterion used in this study is the subjective evaluation made by the clinician; therefore, this result could be attributed to a restrictive application of the UCLA criteria (31) by clinicians when evaluating patients for this research.

Overall, in light of these results, it can be concluded that RAS is an effective measure capable of providing useful feedback on individual progress for users of mental health services and professionals alike. Given the subjectivity of the concept of recovery, it is also worth remarking that many of the tools currently available lack cross-cultural applicability, since they have been developed mainly in Anglo-American contexts (56). Besides, in Italy the perspective of service users is still only marginally taken into account: priority is indeed given to the tools compiled by professionals. The validation of the Italian version of the RAS helps instead to bridge that gap, as it suggests that it would be helpful to combine both perspectives.

Recovery is both a result and a process in which recovery “from” and “in the” mental illness are fluid concepts that are not mutually exclusive (Davidson, 2007). RAS is proving to be one of the best available measures of personal recovery (57). Through this tool, it is possible to assess one’s perceived ability to cope with mental illness and its consequences, as well as the level of self-confidence about the ability to lead a full and satisfying life, notwithstanding and regardless of the severity of the disorder. RAS also allows mental health professionals to better identify the specific areas where interventions can be targeted, in order to effectively improve the health and well-being of their patients. Knowledge of the recovery process can provide service users and their family with the means to overcome the lack of hope, their reliance on professional services, and the loss of control over their lives, perceived as influencing elements of, and associated to, the disorder as well as the symptoms (58). In conclusion, to achieve a proper match between evidence-based techniques and participatory research (59), the concept of recovery is fundamental in order to have services promoting interventions oriented in the same direction of what service users consider to be essential on their road towards healing/recovery.

## SIR 2 Group

### Coordinator Centers

Department of Mental Health, Legnago (VR), Aulss 21, Ileana Boggian, Dario Lamonaca, Tommaso Maniscalco, Silvia Merlin, Laura Barbieri, Katia Bernardelli, Anna Boggian, Alessandra Palmieri, Claudia Menegazzi, Chiara Dal Cero, Piccione Gabriella, Violetta Saggioro, Sotirios Balanikas, Valeria Raffaelli Center For Psychiatric Rehabilitation “Gelmini”, Salorno BZ (Alessandro Svettini, Petra Zambelli, Silvia Bridi, Sabrina Doimo.

### Participant Centers

Center for Psychiatric Rehabilitation “Grieserhof” (BZ) (Luigi Basso, Roberto Tovazzi), Center of Mental Health Rimini (Riccardo Sabatelli, Andrea Parma), Department of Mental Health Padova AULSS 16 (Gianfranco Cuccato, Alessandra Capani, Luca Balboni, Alexandra Baggio, Mario Degli Stefani), Department of Mental Health Roma (Josè Mannu, Raffaella Musillo), Mental Health Service Bassano (VI) AULSS 3, Department of Mental Health (Ruggero Brazzale, Barbara Garbo), Center of Mental Health Arzignano (VI) AULSS 5 (Stefano Zanolini, Laura Andolfo, Alessandra Belfontali, Ileana Rodofile, Roberta Tessari, Jessica Geremia, Flavio Franceschi, Laura Lizza, Jennifer Montagnoli), Mental Health Service Campobasso (Franco Veltro, Antonio Barrea, Alessia Pica, Irene Pontarelli), Service of Mental Health Acquaviva delle Fonti, Department of Mental Health AUSL Bari 3 (Domenico Semisa, Patrizia Fracchiolla, Anna Maria Lerario), Day Center Puntoacapo Coop. Insieme si può, Conegliano (TV) AULSS 9 (Stefania Campana, Mariella Durante), Center of Mental Health Genova ASL 3 (Paolo Peloso, Alessandra Polimo, Simona Gotelli, Lucia Valentini), Department of Mental Health Fano Area Vasta Marche Nord (Lorenza Marinozzi), Niguarda Hospital Milano, Department of Mental Health Niguarda Hospital, Sec. Psychiatry 1 (Stefania Benintende, Maria Frova), Psychiatry Unit IRCCS Centro San Giovanni di Dio, FBF, Brescia (Giuseppe Rossi, Roberta Rossi, Giulia Signorini), Coop Sociale Città Solidale Brindisi (Filomena Maffullo), Department of Clinical Neuroscience, Suore Ospedaliere, FoRiPsi, Albese Con Cassano, Como (Giampaolo Perna, Giovanna Vanni, Daniela Caldirola, Wilma Micieli, Achille Motta, Maddalena Pinti, Paola Noseda, Chiara Piazza), Il Portico Società Cooperativa Sociale Padova (Fabrizio Panozzo, Debora Leardini), Department of Mental Health Bari, Center of Mental Health Brindisi Br/1, Center of Mental Health 4 Altamura Department of Psychiatry ASL Bari (Pietro Nigro, Paola Clemente, Tiziana De Donatis, Marialisa Moramarco), Department of Mental Health ASL 18 Badia Polesine (RO) (Enrica Miriam Rossi, Vanda Bui, Flavia Aldi), Mental Health Center Cuneo (Ugo Palomba), Department of Mental Health, Mental Health Unit 68 ASL Pontecagnano FAIANO (SA) (Giulio Corrivetti, Carmine Martino, Gaetano Pinto), Department of Mental Health Lecce e Sub-unit of San Cesario (Maria Rosaria Lapenna, Serafino De Giorgi, Tiziana De Donatis, Paola Calò, Massimo Viola, Fabiola Mengoli, Irene Tondi, Maria P. Fontana).

## Data Availability Statement

The raw data supporting the conclusions of this manuscript will be made available by the authors, without undue reservation, to any qualified researcher.

## Ethics Statement

Ethical review and approval was not required for the study on human participants in accordance with the local legislation and institutional requirements. The patients/participants provided their written informed consent to participate in this study.

## Author Contributions

RL, IB, DL, and AS contributed to the conception and design of the study. MG and GB organized the database and performed the statistical analysis. DL wrote the first draft of the manuscript. SM, KB, and LB wrote sections of the manuscript. All authors contributed to manuscript revision, and read and approved the submitted version.

## Conflict of Interest

The authors declare that the research was conducted in the absence of any commercial or financial relationships that could be construed as a potential conflict of interest.

## Appendix 1 Ras – Recovery Assessment Scale – (W. Corrigan, M. Salzer, R. Ralph, Y. Sangster, L. Keck, 2004)

**Table:**

**R.A.S.**	
**SCALA DI VALUTAZIONE del RECOVERY**	
**NOME** _______________________________________	**DATA** ______________

Troverà ora alcune affermazioni che descrivono come a volte le persone si sentono rispetto a se stessi e alla propria vita.

Per favore legga con attenzione ogni frase e indichi la risposta che descrive al meglio il grado in cui Lei è d’accordo o meno con quell’affermazione. Indichi per ogni frase se Lei è:

completamente in disaccordo (1), in disaccordo (2), non è sicuro (3), d’accordo (4), o è completamente d’accordo (5).

**Table:**

	Completamente in disaccordo	In disaccordo	Non è sicuro	D’accordo	Completamente d’accordo	NR
1. Ho il desiderio di farcela	1	2	3	4	5	
2. Ho un mio progetto su come arrivare o continuare a star bene	1	2	3	4	5	
3. Ho degli obiettivi nella vita che voglio raggiungere	1	2	3	4	5	
4. Credo di poter raggiungere i miei attuali obiettivi	1	2	3	4	5	
5. Ho uno scopo di vita.	1	2	3	4	5	
6. Anche se non m’importa di me stesso, so che altre persone si interessano a me.	1	2	3	4	5	
7. Capisco come controllare i sintomi della mia malattia mentale.	1	2	3	4	5	
8. Se mi ammalo di nuovo sono in grado di gestire la situazione.	1	2	3	4	5	
9. Sono in grado di identificare i fattori scatenanti i sintomi della mia malattia mentale.	1	2	3	4	5	
10. Sono in grado di aiutare me stesso a stare meglio.	1	2	3	4	5	
11. La paura non m’impedisce di vivere nella maniera che voglio io.	1	2	3	4	5	
12. So che ci sono dei servizi di salute mentale che mi aiutano.	1	2	3	4	5	
13. Ci sono cose che io posso fare per affrontare i sintomi non voluti.	1	2	3	4	5	
14. Sono in grado di gestire ciò che succede nella mia vita.	1	2	3	4	5	
15. Mi piaccio	1	2	3	4	5	
16. Se le persone mi conoscessero veramente, io piacerei loro.	1	2	3	4	5	
17. Sono una persona migliore ora rispetto a prima della mia esperienza di malattia mentale.	1	2	3	4	5	
18. Anche se i miei sintomi possono peggiorare, so di poterli gestire.	1	2	3	4	5	
19. Se continuo a impegnarmi, continuerò a star meglio.	1	2	3	4	5	
20. Io ho idea di chi voglio diventare.	1	2	3	4	5	
21. Le cose accadono per una ragione precisa.	1	2	3	4	5	
22. Alla fine succederà qualcosa di buono.	1	2	3	4	5	
23. Io sono la persona maggiormente responsabile del mio miglioramento.	1	2	3	4	5	
24. Ho speranze per il mio futuro.	1	2	3	4	5	
25. Continuo ad avere nuovi interessi.	1	2	3	4	5	
26. Divertirsi è importante.	1	2	3	4	5	
27. Affrontare la mia malattia mentale non è più il mio principale obiettivo di vita.	1	2	3	4	5	
28. I miei sintomi interferiscono sempre meno con la mia vita.	1	2	3	4	5	
29. Ogni volta che si ripresentano, i miei sintomi sembrano essere un problema per periodi sempre più brevi.	1	2	3	4	5	
30. So quando è il momento di chiedere aiuto.	1	2	3	4	5	
31. Sono disposto a chiedere aiuto.	1	2	3	4	5	
32. Chiedo aiuto quando ne ho bisogno.	1	2	3	4	5	
33. Per me è importante essere in grado di lavorare.	1	2	3	4	5	
34. So cosa mi aiuta a stare meglio.	1	2	3	4	5	
35. Posso imparare dai miei errori.	1	2	3	4	5	
36. Sono in grado di gestire lo stress.	1	2	3	4	5	
37. Ho delle persone su cui posso contare.	1	2	3	4	5	
38. Sono in grado di identificare i segni precoci di ricaduta della mia malattia.	1	2	3	4	5	
39. Anche se non credo in me stesso, altre persone invece sì.	1	2	3	4	5	
40. È importante avere amici di diverso tipo.	1	2	3	4	5	
41. È importante avere abitudini di vita sane.	1	2	3	4	5	
Punteggio totale ____________ Risposte/41						

Il punteggio per ciascuno dei diversi fattori è dato sommando i punteggi degli item tra parentesi

____________ Fiducia in se stessi e speranza (11,14,15,16,20,22,24,25,36)

____________ Disponibilità a chiedere aiuto (30,31,32)

____________ Essere orientati a obiettivi e al successo (1,2,3,4,5)

____________ Fiducia negli altri (6,37,39,40)

____________ Non sentirsi dominati dai sintomi (27,28,29)

**Note per la compilazione:**

La RAS è una scala autosomministrata, ma si consiglia comunque un’autocompilazione asssistita da parte dell’operatore, soprattutto per verificare che sia stato colto il significato della domanda. Se il soggetto non risponde barrare con una X la casella corrispondente alla colonna NR (non risposto).

N.B.

1. Gli item 6, 16 e 39 prevedono un’ipotesi iniziale (anche se non m’importa di me stesso…; se le persone mi conoscessero veramente…; anche se non credo in me stesso…). Il senso della domanda è soprattutto nella seconda parte (…altre persone si interessano a me; …io piacerei loro;…altre persone invece sì), per cui va valutato quanto il soggetto concorda con l’affermazione appunto della seconda parte.
2. Gli item 7, 9 e 27 fanno riferimento esplicito alla malattia mentale. Per i soggetti che negano tale condizione come riferita a se stessi, si può fare invece riferimento al concetto di **stress**. In caso di ulteriore negazione va segnato **NR** (non risposto).
